# Digital conscience in midwifery students: a qualitative study

**DOI:** 10.1590/1806-9282.20260920

**Published:** 2026-11-02

**Authors:** Sumeyye Kose, Rojda Bayar Yıldırım, Sıbel Aydın

**Affiliations:** 1 Midwifery Pendik District Health Directorate, Department of Cancer – Istanbul, Türkiye.; 2 Antalya Bilim University, Faculty of Health Sciences, Department of Midwifery – Antalya, Türkiye.; 3 Health Sciences University, Umraniye Training and Research Hospital, Department of Obstetrics and Gynecology – Istanbul, Türkiye.

**Keywords:** Midwifery, Students, Ethics, Privacy, Digital technology

## Abstract

**OBJECTIVE:**

The aim of this study was to explore midwifery students’ perceptions of digital technology use in educational and clinical settings, their awareness of ethical issues related to digital environments, and their suggestions for preventing ethical violations.

**METHODS:**

This qualitative exploratory study was conducted in a training and research hospital in Türkiye between March and April 2026. Sixteen third- and fourth-year midwifery students were selected through purposive sampling. Data was collected through face-to-face semi-structured interviews and analyzed using thematic analysis.

**RESULTS:**

Participants reported that digital technologies contributed positively to learning by facilitating access to information, strengthening theoretical knowledge, and improving clinical skills. However, they also emphasized ethical concerns regarding the unauthorized sharing of patient information and photographs, breaches of privacy, and potential legal responsibilities associated with digital technology use. Students highlighted the importance of ethics and privacy education, legal regulations, institutional policies, and individual responsibility in preventing ethical violations in digital environments.

**CONCLUSION:**

Midwifery students generally demonstrated positive attitudes toward digital technology use while recognizing the ethical challenges associated with digital environments. Strengthening digital conscience awareness through education, institutional support, and legal frameworks may promote ethical digital practices, protect patient privacy, and support professional responsibility in midwifery care.

## INTRODUCTION

Digital technologies have become an essential component of healthcare education and clinical practice. Mobile devices, digital learning platforms, artificial intelligence applications, and online resources facilitate access to information, support learning processes, and contribute to the development of clinical competencies among healthcare students^1^. In addition, increasing levels of digital literacy among health sciences students have been associated with improved learning experiences and clinical decision-making skills^2^. Previous studies have demonstrated that innovative digital technologies and e-learning approaches enhance educational outcomes and provide effective support for health professions education^3,4^. Despite these advantages, the integration of digital technologies into educational and clinical environments has introduced important ethical challenges related to privacy, confidentiality, data security, and professional conduct. The use of digital data and online platforms may create concerns regarding personal data sharing, informed consent, and ethical decision-making processes^5^. Furthermore, the unauthorized sharing of patient information, photographs, or clinical experiences through digital platforms may result in privacy violations, professional misconduct, and legal consequences^6,7^. Digital conscience is defined as the ability to use digital technologies in a critical, ethical, and responsible manner. It involves recognizing the risks and opportunities of digital environments, protecting privacy and confidentiality, avoiding misinformation, promoting respectful online communication, and maintaining a balanced use of technology while upholding professional and ethical standards^8,9,10^. Professional ethics constitutes a fundamental component of midwifery education and practice. Respect for patient dignity, confidentiality, privacy, and professional responsibility are core elements of quality midwifery care. However, previous studies have reported that midwifery students frequently encounter ethical dilemmas during clinical practice and may require additional support in managing ethical challenges^11,12^. Moreover, a recent scoping review highlighted the importance of strengthening digital professionalism among nursing and midwifery students and emphasized the need for greater awareness regarding the ethical use of social media and digital platforms^13^. In this study, digital conscience refers to awareness of ethical responsibility, privacy protection, professional values, and responsible conduct in digital environments. Although digital ethics and professionalism have been studied among healthcare students, evidence regarding digital conscience awareness among midwifery students remains limited. Therefore, this study aimed to explore midwifery students’ perceptions of digital technology use, awareness of ethical issues in digital environments, and suggestions for preventing ethical violations.

## METHODS

### Aim and study design

This study aimed to explore midwifery students’ perceptions and experiences regarding the use of digital technologies in education and clinical practice, their awareness of ethical issues associated with digital technology use, and their suggestions for preventing ethical violations. A qualitative exploratory design was adopted to obtain an in-depth understanding of participants’ experiences and perspectives.

### Research questions

The study was guided by the following research questions:

1. How do midwifery students perceive the use of digital technologies in education and clinical practice?
2. What ethical issues do midwifery students associate with the use of digital technologies?
3. What are midwifery students’ recommendations for preventing ethical violations related to digital technology use in clinical settings?

### Study variables

As a qualitative study, no dependent or independent variables were defined. The primary areas of inquiry included participants’ experiences with digital technologies, perceptions of ethical and privacy-related concerns, awareness of professional responsibility, and suggestions for the prevention of ethical violations.

### Study setting and duration

The study was conducted in a training and research hospital in Turkey between March and April 2026.

### Population and sample

The study sample was determined using criterion sampling, one of the purposive sampling methods^14^. Participants were third- and fourth-year midwifery students enrolled in the midwifery program of an university in Istanbul, who were undertaking clinical training and met the inclusion criteria. In addition, snowball sampling was used to facilitate participant recruitment, whereby the initial participants referred other eligible students. The sample size was determined according to the principle of data saturation, and data collection continued until no new themes or sub-themes emerged^15^. Accordingly, the study was completed with 16 participants.

### Inclusion and exclusion criteria

Eligible participants were third- and fourth-year undergraduate students enrolled in the midwifery program of a university in Istanbul who were actively participating in clinical training, met the inclusion criteria, and provided written informed consent. Students who were not participating in clinical training during the study period, declined to participate, or had communication difficulties were excluded from the study.

### Ethical approval

Ethical approval was obtained from the Clinical Research Ethics Committee of Istanbul Umraniye Training and Research Hospital (Decision No: 2026/66, Date: February 12, 2026). The study was conducted in accordance with the principles of the Declaration of Helsinki. Written and verbally informed consent was obtained from all participants prior to data collection.

### Data collection instruments

Data was collected using two instruments:

Personal Information Form: Developed by the researchers to collect sociodemographic characteristics and information regarding participants’ digital technology use.

Semi-Structured Interview Form: Developed following a review of the relevant literature on digital ethics, digital technology use, and patient privacy^3,4,5^. The interview guide was reviewed by a panel of 10 experts, including five experts in midwifery and five experts specializing in nursing ethics, to evaluate its content relevance, clarity, and comprehensiveness. Based on their feedback, minor revisions were made to improve the wording and organization of several questions. The final interview guide consisted of open-ended questions exploring perceptions of digital technology use, ethical concerns, professional responsibility, and strategies for preventing ethical violations.

### Study procedure

Eligible participants were informed about the purpose and procedures of the study and invited to participate voluntarily. Data was collected through face-to-face semi-structured interviews conducted in a private environment. Interviews lasted approximately 30–35 min and were audio-recorded with participants’ permission. All recordings were transcribed verbatim immediately after the interviews. To protect confidentiality, participants were assigned numerical identifiers (P1–P16).

### Statistical analysis

Data was analyzed using the six-phase thematic analysis approach proposed by Braun and Clarke^16,17^. First, audio recordings were transcribed verbatim and reviewed repeatedly to achieve familiarity with the data. Meaningful units were then coded inductively, and similar codes were grouped into themes and sub-themes. To enhance analytical rigor and credibility, two researchers independently reviewed the transcripts and coding structure and discussed emerging themes until consensus was achieved.

The trustworthiness of the findings was supported through expert review of the interview guide, pilot testing with five midwifery students to assess the clarity and comprehensibility of the interview questions, independent coding by two researchers, and the inclusion of direct participant quotations. Reporting of the study was guided by the Consolidated Criteria for Reporting Qualitative Research (COREQ) checklist^18^.

## RESULTS

The study included 16 midwifery students aged 20–23 years. Most participants were in the third or fourth year of study and reported regular use of digital technologies and educational platforms.

### Perceptions and experiences of midwifery students regarding the use of digital technologies

Content analysis revealed four main themes and four sub-themes regarding the use of digital technologies in midwifery education and clinical practice. Participants reported that digital technologies reinforced theoretical knowledge, supported clinical skill development, facilitated access to information, and increased professional awareness (Table 1). As P1 stated, “I had never observed a birth before; I first watched it online and prepared myself.” Participants also emphasized that simulation applications and digital resources enhanced clinical competence. However, concerns were raised regarding misinformation and the inappropriate use of digital technologies. As P8 noted, “Only experts in the field should share content.”

**Table 1.** Participants’ perceptions of digital technologies in midwifery education and professional practice.

Theme	Sub-theme	Participants	Frequency	Sample participant quotation
Contribution to learning	Reinforcement and concretization of theoretical knowledge	1, 3, 4, 8, 10, 12, 13, 14, 15	9	P4: “Visual content makes theoretical knowledge more concrete, and the learning process becomes more effective.”
Developing practical skills and clinical competence	Developing practical skills and clinical competence	1, 3, 6, 7, 8, 12, 13, 15	8	P15: “Simulation applications allow us to learn in cases where we cannot observe sufficient cases during clinical practice.”
Accessing and dissemination of knowledge	Increasing education and awareness	2, 5, 9, 11, 16	5	P2: “Training can reach a much larger number of people, and people become more aware of these issues.”
Limitations in use	Controlled and conscious use of digital technologies	8, 10, 12, 13	4	P8: “Only experts in the field should share content because misinformation can be confusing.”

### Midwifery students’ professional ethical perceptions and experiences regarding the use of digital technologies

Content analysis identified one main theme and three sub-themes related to professional ethical perceptions and experiences regarding digital technologies in midwifery practice. Participants identified privacy violations and unauthorized sharing of patient information and photographs as the primary ethical concerns. They emphasized the importance of protecting confidentiality, obtaining consent, and maintaining professional responsibility in digital environments (Table 2). As P13 stated, “Any sharing related to a patient through digital technologies without the patient’s consent constitutes an ethical violation.” Participants also highlighted potential legal consequences of privacy breaches, while a few expressed uncertainties regarding the ethical implications of digital technology use.

**Table 2.** Professional ethical perceptions and experiences regarding the use of digital technologies in midwifery practice.

Theme	Sub-theme	Participants	Frequency	Sample participant quotation
Digital technologies and ethical/privacy issues	Unauthorized sharing of patient information and photographs	1, 3, 5, 7, 8, 10, 11, 12, 13, 14, 15, 16	12	P13: “Any sharing related to a patient through digital technologies without the patient’s consent constitutes an ethical violation.”
	Professional responsibility and potential legal/ethical risks	4, 6, 8, 15, 16	4	P15: “Violation of patient privacy in the use of digital technologies is an important issue... This may expose us to legal sanctions in our profession.”
	Uncertainty and indecision	2, 9	2	P9: “I am undecided about this; I do not fully know whether digital technologies create ethical or professional responsibility issues.”

### Solution suggestions for ethical issues in the use of digital technologies in midwifery practice

Analysis revealed four main themes and four sub-themes regarding solution suggestions for ethical issues related to digital technologies in midwifery practice. Participants emphasized education, legal regulations, institutional policies, and individual responsibility as key strategies for preventing ethical violations. Education was the most frequently recommended approach (Table 3). As P16 stated, “Education should be provided especially in schools on this issue.” Participants also highlighted the need for legal sanctions, institutional safeguards, and greater ethical awareness.

**Table 3.** Participants’ views on solution suggestions for ethical issues in the use of digital technologies.

Theme	Sub-theme	Participants	Frequency	Sample participant quotation
Education and awareness	Increasing ethics and privacy education	1, 2, 4, 6, 7, 8, 9, 10, 13, 16	10	P16: “Education should be provided especially in schools on this issue… In addition, informative training sessions can be organized in hospitals before starting clinical internships.”
Legal regulation and supervision	Legal sanctions and monitoring mechanisms	1, 3, 4, 5, 6	5	P4: “Regular inspections can be conducted, and frequent ethics training can be provided.”
Institutional regulations and technological measures	Institutional policies and development of digital systems	6, 12, 13, 14, 15	5	P13: “Institutions should introduce specific regulations regarding the use of digital technologies and ensure that this use is carried out within an authorized framework.”
Individual responsibility	Empathy, adherence to ethical principles, and conscious behavior	7, 11, 15	3	P11: “I believe that change should start from the individual… we should act in accordance with ethical principles and practice empathy.”

## DISCUSSION

This study explored midwifery students’ perceptions of digital technologies and related ethical issues. Overall, participants viewed digital technologies as valuable tools for learning and professional development while simultaneously recognizing the ethical challenges associated with their use.

### Perceptions and experiences regarding the use of digital technologies

Participants reported that digital technologies supported learning by reinforcing theoretical knowledge, facilitating access to information, and enhancing clinical competence. Students emphasized the value of digital resources, simulation applications, and online educational content for clinical preparation. These findings are consistent with studies showing that digital learning tools improve clinical competencies, problem-solving, and decision-making skills among healthcare students^19,20,21^.

Similar findings have shown that digital systems facilitate access to information and support learning during clinical placements^22^. However, some participants expressed concerns regarding misinformation and uncontrolled technology use, highlighting the importance of responsible digital engagement. These findings suggest that digital technologies can enhance midwifery students’ preparedness for clinical practice when combined with critical thinking and ethical awareness. Therefore, integrating digital literacy and digital professionalism into midwifery education may contribute to the safe, evidence-based, and ethical use of digital technologies in future clinical practice.

### Professional ethical perceptions and experiences regarding the use of digital technologies

The most prominent ethical concern was the unauthorized sharing of patient information and photographs. Participants emphasized confidentiality, informed consent, and professional responsibility in digital environments. Similar concerns regarding confidentiality breaches, professional boundaries, and limited awareness of legal responsibilities have been reported previously^23,24^. Therefore, fostering digital conscience during midwifery education may strengthen future midwives’ ability to critically evaluate digital information, protect patient confidentiality, and use digital technologies ethically in clinical practice. This interpretation is consistent with the existing literature^25,26^.

### Solution suggestions for ethical issues in the use of digital technologies

Participants identified education, legal regulation, institutional support, and individual responsibility as key strategies for preventing ethical violations. Education and awareness were the most frequently emphasized recommendations. Previous studies similarly underline the importance of ethics education, digital ethics guidelines, and regulatory frameworks in reducing ethical risks associated with digital technologies^11,27,28^. These findings suggest that the ethical use of digital technologies requires educational, institutional, and regulatory support.

Integrating these strategies into midwifery education through structured curricula, simulation-based learning, institutional digital ethics policies, and legal and ethical training may better prepare students for the responsible use of digital technologies in clinical practice. This approach is consistent with the existing literature highlighting the value of educational technologies and structured educational interventions in strengthening professional competencies among future healthcare professionals^29,30^.

### Limitations and strengths

The qualitative design, small sample size (n=16), and single-institution setting may limit the transferability of the findings. In addition, because the findings were based on participants’ self-reported experiences, responses regarding ethical behavior, patient confidentiality, and professional responsibility may have been influenced by social desirability bias. Nevertheless, the study has several important strengths. The qualitative design enabled an in-depth exploration of midwifery students’ perceptions and experiences regarding digital conscience, generating rich and context-specific data that could not be captured through quantitative methods. The use of semi-structured, in-depth interviews facilitated a comprehensive understanding of participants’ perspectives, while expert review of the interview guide, pilot testing, and independent coding by two researchers enhanced the credibility and trustworthiness of the findings. Furthermore, reporting the study in accordance with the COREQ guidelines strengthened the methodological transparency and rigor of the research. The findings also contribute to the limited qualitative literature on digital conscience in midwifery education and provide evidence that may inform future educational strategies and professional practice.

## CONCLUSION

Midwifery students viewed digital technologies as valuable for learning and clinical skill development but also recognized ethical challenges related to privacy, confidentiality, and professional responsibility. Strengthening digital ethics awareness through education and institutional support may promote the responsible use of digital technologies and help protect patient rights.

## Data Availability

The datasets generated and/or analyzed during the current study are available from the corresponding author upon reasonable request.
